# On the importance of primary and community healthcare in relation to global health and environmental threats: lessons from the COVID-19 crisis

**DOI:** 10.1136/bmjgh-2020-004111

**Published:** 2021-03-10

**Authors:** Paolo Lauriola, Piedad Martín-Olmedo, Giovanni S. Leonardi, Catherine Bouland, Robert Verheij, Michel L A Dückers, Martie van Tongeren, Ferdinando Laghi, Peter van den Hazel, Ozden Gokdemir, Evelyn Segredo, Ruth A Etzel, Alan Abelsohn, Fabrizio Bianchi, Roberto Romizi, Giuseppe Miserotti, Francesco Romizi, Paolo Bortolotti, Emanuele Vinci, Guido Giustetto, Mariagrazia Santamaria, Alice Serafini, Samantha Pegoraro, Raymond Agius, Ariana Zeka

**Affiliations:** 1 International Society of Doctors for the Environment (ISDE-Italy), Modena, Italy; 2 Escuela Andaluza de Salud Pública, Granada, Spain; 3 Department of Public Health, Environments and Society, London School of Hygiene and Tropical Medicine, London, UK; 4 ULB-Ecole de Santé Publique, Bruxelles, Belgium; 5 Netherlands Institute for Health Services Research (NIVEL), Utrecht, The Netherlands; 6 Centre for Occupational and Environmental Health, University of Manchester, Manchester, UK; 7 International Society of Doctors for the Environment (ISDE-Int’l), Geneva, Switzerland; 8 International Network on Children’s Health, Environment and Safety (INCHES), Ellecom, The Netherlands; 9 Faculty of Medicine, Izmir University of Economics, Balçova/Izmir, Turkey; 10 Uruguayan Society of Family and Community Medicine, Montevideo, Uruguay; 11 Milken Institute School of Public Health, The George Washington University, Washington, DC, USA; 12 Department of Family and Community Medicine, University of Toronto, Toronto, Ontario, Canada; 13 Istituto Fisiologia Clinica, Consiglio Nazionale della Ricerca (CNR-IFC), Pisa, Italy; 14 International Society of Doctors for the Environment (ISDE-Italy), Arezzo, Italy; 15 International Society of Doctors for the Environment (ISDE-Italy), Piacenza, Italy; 16 Medical Order Trento, Trento, Italy; 17 Health and Environment Working Group, National Medical Orders Federation, Rome, Italy; 18 National Medical Orders Federation, Rome, Italy; 19 Local Health Authority, Foggia, Italy; 20 Local Health Authority, Modena, Italy; 21 Italian Climate Network, Milano, Italy; 22 Institute of Environment, Health and Societies, Brunel University London, Uxbridge, UK

**Keywords:** environmental health, health systems, public health, prevention strategies, health policy

## Abstract

In the course of the COVID-19 pandemic, it has become clear that primary healthcare systems play a critical role in clinical care, such as patient screening, triage, physical and psychological support and also in promoting good community advice and awareness in coordination with secondary healthcare and preventive care. Because of the role of social and environmental factors in COVID-19 transmission and burden of disease, it is essential to ensure that there is adequate coordination of population-based health services and public health interventions. The COVID-19 pandemic has shown the primary and community healthcare (P&CHC) system’s weaknesses worldwide. In many instances, P&CHC played only a minor role, the emphasis being on hospital and intensive care beds. This was compounded by political failures, in supporting local community resilience. Placing community building, social cohesion and resilience at the forefront of dealing with the COVID-19 crisis can help align solutions that provide a vision of ‘planetary health’. This can be achieved by involving local well-being and participation in the face of any pervasive health and environmental crisis, including other epidemics and large-scale ecological crises. This paper proposes that P&CHC should take on two critical roles: first, to support local problem-solving efforts and to serve as a partner in innovative approaches to safeguarding community well-being; and second, to understand the local environment and health risks in the context of the global health perspective. We see this as an opportunity of immediate value and broad consequence beyond the control of the COVID-19 pandemic.

**Summary box**

- In 1978, the Alma-Ata (Kazakhstan) Declaration asserted the strategic importance of high-quality primary healthcare in the creation of effective and responsive healthcare systems; unfortunately, 42 years later, this vision has not been attained yet.
- In October 2018, health experts and policymakers met in Astana (Kazakhstan) to renew the commitment for comprehensive available healthcare to all, reaffirming the commitments to the Alma-Ata core principles.
- The lack of involvement of the primary and community healthcare (P&CHC) systems during the COVID-19 pandemic has highlighted their crucial role in clinical and public health functions such as diagnosis (screening), triage, contact tracing and in the short-term and long-term physical and psychological monitoring and management of patients.
- The current emergency has also shown the need for P&CHC to work in coordination with other healthcare and public health organisations at the community level.
- There is an urgent need for environmental public health policies with an intersectoral and global perspective acknowledging the influence of the environment and the need to act at the community level.
- Besides the traditional focus on the individual, it is essential that public health agencies encourage and promote the involvement of P&CHC within the framework of community and environmental health.
- The value of this has been highlighted by the response to COVID-19, but it is equally central to the management of other crises affecting communities.

## Introduction and objectives

The novel SARS-CoV-2 and its resultant COVID-19 disease is the most challenging health emergency faced by humanity in living memory. This pandemic represents a grave threat to global health, economy and the social well-being of people.

The COVID-19 pandemic has emphasised the crucial role that primary and community healthcare (P&CHC) systems^1 2^ could play in diagnosis or screening and triage, as well as in the short-term and long-term monitoring of the physical and psychological conditions of those affected. The current emergency has also highlighted the need for P&CHC to work on tracing and management of contacts in coordination with other healthcare organisations such as local and national public health institutions,^3 4^ as well as those at the community level^5^ such as community-based civil society groups and community health workers.

Because of the role of social and environmental factors in COVID-19 transmission and in the burden of disease, it is essential to ensure that population-based health services and interventions are adequately prioritised and coordinated between P&CHC providers and public health organisations.

There are recognisable dimensions to the complex domain of how decisions are made to manage rapidly emerging problems. Each approach to crisis management has its merits, and they are all interconnected.^6^ The role of P&CHC is central to the effective management of any health crisis.

Despite historical and ongoing discrepancies between and within the policy and scientific communities, mainly due to the complexity mentioned above, the pandemic has shown the importance of facilitating the inter-relationship and mutual support between the health, social, environmental and political-economic domains.

The goal of this article is to highlight the crucial role that P&CHC could play in managing the COVID-19 pandemic, in addressing key environmental and social aspects involved in viral transmission, and assist in the design of the interventions to control it. The article discusses how the success of such interventions depends on an overall vision of integrating environmental health with social and healthcare. It makes the case that enhancing such capacity now will have a more comprehensive benefit for better addressing this crisis and others in the future.

We lay emphasis on the importance of a multidisciplinary and effective workforce in environmental public health (EPH),^7^ in collaboration with P&CHC and hospitals.

The COVID-19 pandemic is not the only concern we address with this proposed strategy; this is an exemplar of a ‘stress test’ of global health and environmental threats.

## SARS-CoV-2 and COVID-19: an example of environmental and sociopolitical imbalance

The origin of SARS-CoV-2 and the subsequent COVID-19 pandemic have revealed the precariousness of the systems on which trade, food, energy, transportation and social safety and security nets depend.

The reported origin of the current pandemic emphasises the exceptional relationship that exists between the environment, animal and human health (One Health approach).^8^ Evidence suggests that SARS-CoV-2 emerged from transportation and trading of wild animals in wet markets.^9^ About 1 billion cases of illness and millions of deaths occur every year from zoonoses, which comprise about 60% of all emerging infectious diseases globally.^10^ The majority of these (60.3%) are caused by zoonotic pathogens, out of which 72% have a wildlife origin.^11^ Ecosystem health, wildlife health and human health are increasingly interconnected, leading to an urgent need to develop locally and globally coordinated plans for better addressing the impacts of environmental change on zoonotic and parasitic infections,^12^ including COVID-19.^13^

Local, national, large-scale and systemic environmental changes and disruptions, such as biodiversity loss, disruption of natural habitats, extinction of species, climate crisis, pollution, together with societal and demographic changes (eg, urbanisation, increase in mobility, population ageing), are likely determinants of different epidemics.^14^ Both environmental and social factors play an important role by facilitating the occurrence of new or re-emerging infectious diseases, and/or by greatly modulating their health impacts.

## Global/local, two sides of the same coin

The way in which the COVID-19 crisis developed in time and space made clear that the distinction between local and global has become largely unimportant.^15^ The local ecological wildlife and human interactions in Wuhan, China, have been recognised as the origin of the SARS-CoV-2 spillover, which afterwards quickly spread across the globe. Such local incidents are related to global environmental issues such as ecological erosion and the climate crisis. Regional and local political and economic decisions that promote unsustainable practices lead to deforestation, intensive farming, unsustainable energy production, air pollution and deterioration of water and soil, all of which contribute further to the global climate crisis, increasing the likelihood of emerging global impacts in the future. The role of a globalised economic order that prioritises short-term profits of private interests, which benefit actors in wealthy countries and elites in low-and-middle-income countries (LMICs), while dismissing environmental and social fallout as ‘negative externalities’, are clearly recognised. There are many historical examples of how societies did not recognise the 'circularity in nature' with disastrous results. Such lessons have shown how big businesses affect the environment, and what individuals and communities can do about our environmental problems today.^16^

And while global system-level change is needed, communities can also play an important role in helping to address environmental problems at multiple levels. P&CHC can contribute to addressing such global challenges, with the view of prevention and protection from harm of communities, with global benefits by sharing of experiences and expertise to improve competencies and capabilities at the local level.

These efforts are not at all simple to deploy for many reasons such as the overall organisation or conflicts between science and politics, with some clear examples across the world as provided here 17–19

The uneven distribution of COVID-19 cases and severity of the disease^20^ are determined by socioeconomic circumstances, inequality associated with poor working and living conditions, access to services, mobility and pre-existing health conditions associated with deprivation.^21^ Populations lacking access to health services in normal circumstances are left most vulnerable during times of crisis,^22^ and addressing such social determinants and inequities is part of the challenge of P&CHC at global level and first and foremost at local level.

## P&CHC as a cornerstone to address global issues at the local level

P&CHC has historically had a crucial role in communities dealing with global challenges. Societies are ageing, with many facing increasing rates of obesity, non-communicable diseases (NCDs) and new and re-emerging infectious diseases, with health spending rising.^23^ It is therefore imperative to redesign health systems that contribute to community health by addressing environmental factors.^16^ The rapid spread of COVID-19 added complexity to these challenges. A multidisciplinary P&CHC workforce can play a vital role in recognition and management of environmental and social factors of community health. Family doctors (FDs) and family paediatricians (FPs) are usually the first contact point for patients, even in the current pandemic, especially in high-income countries (see the Low and Middle-Income Countries section for considerations more specific to LMICs).

### Health impacts in different settings of P&CHC approach

The main critical threat of the COVID-19 pandemic was likely due to the potential collapse of the health service. This has been extensively investigated worldwide.^24 25^ The Italian experience is an example.

Italy was the first European country involved in the pandemic and has been one of the worst affected. The first confirmed cases were detected on 31 January 2020. Later, a cluster of cases with the first deaths was identified in -the northern region of Lombardy. The virus had also spread to the neighbouring region of Veneto, but not initially to other areas of the country.

The Italian healthcare system is highly decentralised; different regions implemented different policy responses to COVID-19. The most notable example is the contrast between the approaches adopted by Lombardy and Veneto, two neighbouring regions with similar socioeconomic profiles.

Lombardy, one of Europe’s wealthiest and most industrial productive areas, was disproportionately hit by COVID-19. Veneto, which is similarly wealthy, by contrast, fared significantly better. In the second wave of the pandemic in autumn, Lombardy scored at the highest level of risk.^26^ By the end of December 2020, the situation was changing, arguably linked to many contributing factors (virological, organisational), which are under investigation.

The spread of COVID-19 and the policy measures taken in these two regions were shaped by a multitude of factors, including the different public health management choices applied in each region.^27^ Interventions in Lombardy—like in other regions which experienced much larger case fatality rates—were based mainly on hospital activities.^28^ The region of Veneto, at least in the first half of the year 2020, relied more on the involvement of primary care doctors, with resulting lower health impacts. While the situation is more complex with many factors (such as socioeconomic, organisational, infrastructure, public health surveillance) contributing to the observed differences in health impacts, this experience highlighted the importance of P&CHC as the front line in dealing with health crises.

### Connections of P&CHC

The connection between P&CHC and occupational health (OH) is well established. This is important for better prevention of chronic conditions (such as musculoskeletal or mental health disorders) that lead to absenteeism or early departure from the labour force.^29^ Efforts to improve workers’ physical and mental health include raising awareness among managers, improving the physical working environment, humanising social relations at work and offering programmes dedicated to encouraging disabled people to return to work. Equally important is the health surveillance of workers especially for physical, chemical or biological hazards at work.

In this respect, COVID-19 presents an almost unique instance where occupational and environmental risk factors have overlapped, generating a health, social and economic impact of a magnitude not seen before. Failure of OH control at work will affect many individuals; failure of control in the community will result in many sick workers. Hence, the close coordination between P&CHC, OH, EPH and enforcement authorities is essential.^30^

P&CHC can play two critical roles: one, as partners to public health policymakers and agencies in contributing to monitoring impacts and serving as ‘anchor institutions’^31^ for implementing local solutions and innovative approaches to safeguarding community health; and two, an essential role in community building, social cohesion and resilience.^32^

## P&CHC: the linchpin connecting environment and health

COVID-19 severity and fatality have been shown highest among those made vulnerable by less favourable structural social conditions.^22^ The unequal distribution of health and mortality burden disfavouring those most vulnerable, between and within countries and communities, shows similarities across the spectrum of health conditions—these being communicable or non-communicable.^33 34^

In the case of COVID-19, the term ‘syndemic’ has been cited, as the health burden has been beyond the number of cases of infection, and has extended to several NCDs aspects.^35^ First, biological and social interactions of COVID-19 with underlying non-communicable health conditions often lead to more serious disease progression, including death. Second, services for NCDs have been considerably disrupted in three-quarters of countries globally^36^ with associated impacts. As environmental factors are estimated to account for at least 25% of the preventable burden of disease, including NCDs,^37^ a perspective for primary prevention of community disease burden cannot ignore EPH.

The health effects associated with environmental risk factors, usually quantified through mortality, emergency visits and hospitalisations, are under-represented, as many illnesses treated by FDs or FPs are missed in these numbers.^38^ For example, vectorborne infectious diseases show a geographical heterogeneity linked to climate crisis.^39^ In these cases, global strategies addressing climate crisis can and should be implemented locally. The role of local community-based FDs and FPs is essential in what they can do to encourage mitigation of climate crisis and to assist communities in their adaptation efforts. A clear and comprehensive example of the activities which could be carried out within the P&CHC framework to address global environmental risk factors is provided in box 1.^40^

Box 1 **Primary and community healthcare role in relation to global health threats**

- Public education and raising awareness.
- Day-by-day involvement in local/regional/national strategies to tackle antimicrobial resistance within a One Health approach.
- Early alert systems: impending weather extremes, infectious disease outbreaks.
- Disaster preparedness, including increasing the health system’s ‘surge’ capacity to respond to emergencies.
- Enhanced infectious disease control programmes.
  - Food safety, vaccine programmes, case detection and treatment.
- Improved surveillance.
  - Vector control.
  - Risk indicators (eg, aeroallergen concentration).
  - Health outcomes (eg, infectious disease outbreaks, rural suicides, seasonal asthma peaks).
- Appropriate health workforce training, including continuing professional development (eg, updated understanding of climatic influences on health, training in public health).

(Adapted from Blashki *et al* [40]).

## Implementing stronger P&CHC

New approaches can be implemented as preventive health/care measures with a broad integrated perspective. Action is needed at multiple levels, and which is attuned to different contexts.

### Local-national level

A policy survey conducted by the Organisation for Economic Co-operation and Development (OECD)^1^ suggests that key policies and strategies delivering better P&CHC should be based on:

- New models of care: rather than the single-practice physician, multidisciplinary health teams supported by digital technology should be proactively engaged in preventive care.^41^
- More economic incentives: to encourage P&CHC to work in teams and focus on prevention and continuity of care.
- A broader role for patients, including involving the patients in the co-management of their health.

Digitalisation is essential to integrate P&CHC further at the community level. ‘Precision public health’ is an approach which uses data from traditional and emerging sources, for example, big data to target interventions for populations by person, place and time.^42^ With such approach, supported by strong empirical foundations, electronic health records from P&CHC, especially if in combination with data on environmental circumstances, represent an essential source of data and understanding at the local level.^43^

As such, FDs and FPs could play a crucial role in connecting global concerns with local actions through the information they provide, which can influence evidence-based needed policy, in addition to their important role in community awareness and attitudes.^44^

EPH at the level of P&CHC could be strengthened through agreements with:

1. Employers of primary care staff (usually in the public sector), to modify contracts and job descriptions of selected currently employed P&CHC staff to identify tasks related to EPH aspects of community health.
2. Universities and other agencies required for provision of training of P&CHC staff, to identify appropriate competencies and standards required, and develop related training programmes to enable them to perform the tasks related to EPH aspects of community health. It is possible that a first cohort of P&CHC staff may not have acquired sufficient training in EPH in the absence of formally recognised training programmes, so this aspect will concern successive cohorts; furthermore, lifelong learning would drive towards a larger number.
3. Professional registration bodies, such as Ordine dei Medici/General Medical Council and other professional registration authorities, depending on country and type of profession,^45^ to conduct and conclude negotiations with employers and universities/training agencies for skills and competencies required to achieve registration of staff who have completed training related to EPH aspects of community health, as part of their professional qualification.
4. Health agencies to promote secondary use of electronic health records data from primary and community care facilities and linking that to other data relating to the environment.

### International level

International organisations should provide a framework where the crucial role of P&CHC in EPH is emphasised especially with respect to data sharing, responsibilities, capability and consistent capacity building. The following organisations are already dealing with such issues, and should be encouraged to develop such framework with a collaborative perspective.

The World Health Organisation stated that a vision for primary health care (PHC) in the 21st century must be based on individuals and communities as a unique focus.^46^

The World Organization of Family Doctors is an international organisation that aims at improving the quality of life by fostering high standards of care in general practice/family medicine considering the environment.^47^

Health and Environment Alliance is the leading European not-for-profit organisation addressing how the natural and built environments affect health in Europe.^48^

International Society of Doctors for the Environment was founded 30 years ago, aiming at educating physicians and the general public.^49^

### Low and middle-income countries

Particular attention must be paid to LMICs with populations and economies more vulnerable to the immediate effects of the climate crisis, including zoonotic infections or parasitic disease, and the long-term health, social and economic impacts of the current pandemic, and more generally to the difficulties towards a sustainable and equitable development.

Integration of PHC in view of a community health perspective in LMICs is complex. As such, two points^1^ should be raised:

1. Data: many LMICs lack sufficient data on PHC performance, with some countries lacking even basic vital registration data.^50^
  Data gaps that influence PHC systems can be found at any level and dimension of the health system, including aspects of governance, leadership and population health management.
2. Integration: integrating a PHC system is a combined effort, and there is no single tool to achieve this. The PHC systems design approach must be strengthened, incorporating human resources for health, financing, facilities, private sector and demand to develop and enhance integrated systems.

P&CHC is mainly based on the awareness of the socioeconomic, capacity and cultural/historical features in which the FDs work. As such, according to the Astana Declaration,^51^ a holistic approach must integrate efforts to deal with other primary conditions such as malnutrition, waterborne diseases and infectious diseases which disproportionately impact LMICs.

According to OECD,^1^ in LMICs, attention needs to be directed to:

- District management systems with consistent investments in front-line PHC and community health workers.
- Mobilisation of sufficient financial resources to reduce inequalities in the ability to pay P&CHC services, and to provide financial protection against impoverishment from catastrophic healthcare costs.
- Improved learning between healthcare systems and integrated care, and development of strategies for engaging the private sector.

## Capability and capacity: the role of education

### Background

Knowledge and recognition of EPH issues are not common among physicians and other health practitioners. Physicians, in particular FDs and FPs, should engage with environmental concerns in both their role as clinicians as well as their role as public health practitioners. EPH training of physicians should include knowledge and skills in recognising, diagnosing and treating health problems caused by environmental risk factors. Training would include toxicology and epidemiology, both as basic science, and in the clinical training of FDs before, during and after their specialist training. Education and training of physicians as public health practitioners must include knowledge and skills in general public health, EPH and citizen participation. The successful response of the health system in the Republic of Korea has been attributed to the holistic education of the physicians of that country.^25^

### Proposals and perspectives

The challenge of creating a curriculum that fits a diversity of specialties and levels of training, and covers both the science and implementation of EPH, is not straightforward. However, it is important to be strongly focused on pursuing exactly that.^52^ The overall organisation of this curriculum can be based on an accredited system, according to the different levels and educational needs. It will need to consider flexibly different models of programmes, training and capacity provisions. It will need to be promoted at the highest level of medical schools and universities, as well as in community health training (see the Local-National Level section). Universities will need to collaborate with municipal and regional institutions for community health, in identifying standards for required training, at undergraduate and postgraduate levels, in both clinical and community or public health aspects of environmental health. The integration of academic and practical experience is crucial in the success of training in the environmental aspects of clinical medicine and of EPH at the community level. Education must be inclusive, flexible, ongoing and reflective of changes in societal needs.

COVID-19 illustrates very well how the principle of capacity building founded on modification of tasks for P&CHC staff already in post, as we advocate in the Local-National Level section, may be relevant to deployment of existing staff to work on any public health topic. During the COVID-19 emergency, education of all healthcare staff was effective because most of them were already in post. Staff redeployment to COVID-19 duties was usually accepted without modification of contracts and job descriptions, in view of the acceptance of an obvious emergency. A brief statement requiring tasks to be provided on public health, and in particular EPH, should be included in a contract and job description to support and facilitate effective delivery of such tasks by P&CHC staff.

## Conclusions and recommendations

There are multiple connections between the environment and health. There is an urgent need for EPH policies with an intersectoral and global perspective.

There is a close relationship between planetary fragility, social frailty and poverty; social justice and environmental justice are two sides of the same coin.

The crucial challenge is to move towards a system that is economically viable, ecologically sustainable and socially fair.^53^

In the past, the P&CHC was based on the relationship between doctor and patient. Today, the community dimension of the COVID-19 crisis with its NCDs and social aspects provides a lesson for any pervasive crisis.^33^ The influence of the environment on health and well-being in their acute and chronic manifestations, and the need to act at the community level are increasingly evident.

Because they are embedded in and closely connected to local communities, primary and community care needs to be strengthened to play a more important role in the relation between individual health and the environment. Global issues need to tangibly translate to local awareness in determining local healthcare policies as well as pandemic and other responses, including the provision of essential data and information about local health. More than anyone else in the healthcare system, P&CHC services are the ones that can make the link between people’s health and the environment they live in.

## Data Availability

There are no data in this work.
